# Efficient Rescue of Retinal Degeneration in *Pde6a* Mice by Engineered Base Editing and Prime Editing

**DOI:** 10.1002/advs.202405628

**Published:** 2024-09-19

**Authors:** Zhiquan Liu, Siyu Chen, Alexander E. Davis, Chien‐Hui Lo, Qing Wang, Tingting Li, Ke Ning, Qi Zhang, Jingyu Zhao, Sui Wang, Yang Sun

**Affiliations:** ^1^ Department of Ophthalmology Stanford University School of Medicine Palo Alto CA 94304 USA; ^2^ Department of Ophthalmology Shanghai East Hospital Tongji University School of Medicine Shanghai 200120 China; ^3^ Palo Alto Veterans Administration Palo Alto CA 94304 USA

**Keywords:** base editing (BE), pde6a, prime editing (PE), retinal degeneration

## Abstract

Retinitis pigmentosa (RP) is a complex spectrum of inherited retinal diseases marked by the gradual loss of photoreceptor cells, ultimately leading to blindness. Among these, mutations in PDE6A, responsible for encoding a cGMP‐specific phosphodiesterase, stand out as pivotal in autosomal recessive RP (RP43). Unfortunately, no effective therapy currently exists for this specific form of RP. However, recent advancements in genome editing, such as base editing (BE) and prime editing (PE), offer a promising avenue for precise and efficient gene therapy. Here, it is illustrated that the engineered BE and PE systems, particularly PE, exhibit high efficiency in rescuing a target point mutation with minimal bystander effects in an RP mouse model carrying the *Pde6a* (c.2009A > G, p.D670G) mutation. The optimized BE and PE systems are first screened in N2a cells and subsequently assessed in electroporated mouse retinas. Notably, the optimal PE system, delivered via dual adeno‐associated virus (AAV), precisely corrects the pathogenic mutation with average 9.4% efficiency, with no detectable bystander editing. This correction restores PDE6A protein expression, preserved photoreceptors, and rescued retinal function in *Pde6a* mice. Therefore, this study offers a proof‐of‐concept demonstration for the treatment of *Pde6a*‐related retinal degeneration using BE and PE systems.

## Graphical abstract

This study examines the role of new genome editing tools in treating retinitis pigmentosa (RP), particularly focusing on the *Pde6a*‐D670G mutation. Highlighting the potential of engineered base editing (BE) and prime editing (PE), it demonstrates how these advanced techniques can precisely correct the point mutation in *Pde6a* mouse model, effectively restoring retinal function.

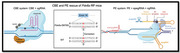

## 1 Introduction

Retinitis pigmentosa (RP) represents a group of inherited retinal disorders characterized by progressive degeneration of photoreceptor cells, ultimately leading to vision loss and blindness.^[^ ^1^ ^]^ RP is the most common form of inherited retinal disease with an estimated incidence of one in 4000 human births, imposing a substantial burden on both individuals and society.^[^ ^1^, ^2^ ^]^ Affected patients present with night‐blindness and progressive constriction of their peripheral visual fields while retaining central vision. Loss of rod photoreceptors is followed by loss of cone photoreceptors, causing an irreversible decline in visual acuity that may lead to blindness.^[^ ^2^, ^3^ ^]^ The *Pde6a* gene encodes the α subunit of rod‐specific cyclic guanosine monophosphate (cGMP)‐phosphodiesterase, a critical enzyme involved in the phototransduction cascade within rod photoreceptor cells.^[^ ^2^ ^]^ Mutations in PDE6A disrupt the function of this enzyme, leading to dysregulation of cGMP levels and subsequent photoreceptor cell death. One such mutation, c.2009A>G (p.D670G), results in an amino acid substitution that destabilizes the PDE6A protein structure, impairing its catalytic activity and contributing to the pathogenesis of RP in a *Pde6a* ^nmf363/nmf363^ mouse model.^[^ ^4^ ^]^ This mouse model accurately simulates the phenotype of human retinal degeneration and is widely used in *Pde6a* gene therapy research.^[^ ^4^, ^5^ ^]^

Despite significant advancements in our understanding of the genetic basis of RP, effective treatments for *Pde6a*‐related RP remain elusive. Traditional gene therapy approaches, such as adeno‐associated virus (AAV) vector‐mediated gene replacement therapy, have shown limited success in treating RP caused by *Pde6a* mutations.^[^ ^2^, ^6^ ^]^ However, recent breakthroughs in genome editing technologies, including base editing (BE) and prime editing (PE), hold promise for precise correction of disease‐causing mutations with minimal side effects.^[^ ^7^ ^]^ Because BE and PE can precisely install targeted point mutations without requiring DNA double strand breaks (DSBs) or donor templates, they are better suited for precise gene correction than other genome editing techniques. Cytidine base editors (CBEs), composed of a cytidine deaminase fused to Cas9 nickase (nCas9, D10A), enable the conversion of C·G to T·A base pair in the target site.^[^ ^8^ ^]^ In addition, PE involves the fusion of a Cas9 nickase (nCas9, H840A) with a reverse transcriptase (RT) and a PE guide RNA and provides the most versatile tool to precisely introduce not only all types of transitions and transversions but also small insertions or deletions.^[^ ^9^ ^]^ BE and PE offer the possibility of treating a wide range of genetic diseases by directly editing the patient's DNA in a precise and targeted manner.

In this study, we aimed to use BE and PE technologies to correct *Pde6a* (c.2009A > G, p.D670G) mutation in an RP mouse model. We demonstrated the efficacy of optimized BE and PE systems in correcting the pathogenic mutation and preserving retinal photoreceptors. Notably, the dual‐AAV delivered PE system demonstrates remarkable precision, devoid of unwanted bystander mutations, and effectively restores retinal function in the *Pde6a* mouse model. Our findings provide valuable insights into the development of novel therapeutic strategies for *Pde6a*‐related RP and highlight the potential of BE and PE technologies in the treatment of inherited retinal disorders.

## 2 Results

### 2.1 Design and Optimization of the CBE System for *Pde6a* Mutation Correction

**Figure 1:**
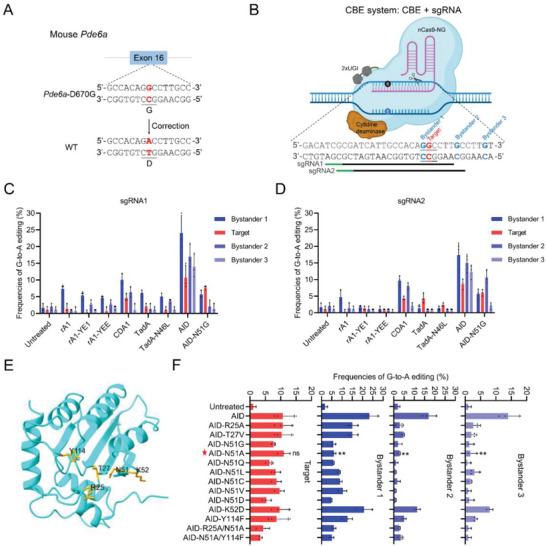
In vitro screening of the optimal CBE system for *Pde6a* mutation correction. A) Schematic representation of ideal *Pde6a* (c.2009A > G, p.D670G) mutation correction. B) Schematic representation of CBE‐sgRNA design for correcting *Pde6a* mutation. Target base, red; potential bystander bases, blue; PAM sequence, green. The schematic diagram was created by biorender.com. C,D) Target and bystander editing efficiencies of the eight tested CBE systems with sgRNA1 (C) or sgRNA2 (D) in N2a cells. (*n* = 3 biologically independent experiments). E) Schematic diagram of the structure of AID deaminase (Protein Data Bank accession: 5W1C). The five residues (R25, T27, N51, K52, and Y114) that may interact with the deaminated cytidine are highlighted in yellow. F) Comparison of the target and bystander editing efficiency of AID and AID variants at the *Pde6a* mutation site in N2a cells. (*n* = 3 biologically independent experiments). Two tailed unpaired t‐tests, compared to AID group. **p < 0.01, ns: non‐significant difference.

The *Pde6a* ^nmf363/nmf363^ mice (hereafter termed *Pde6a* mice), which carry a missense mutation (c.2009A > G, p.D670G) in the *Pde6a* gene and exhibit moderate photoreceptor degeneration, has been widely used to study *Pde6a*‐related RP (**Figure** **1**A).^[^ ^4^ ^]^ The CBE system can achieve G‐to‐A base conversions within the editing window under appropriate sgRNA guidance, making it suitable for repairing this A‐to‐G mutation (Figure 1B). However, we noted the presence of multiple adjacent Gs around the target G, particularly the proximal bystander 1, which greatly predisposes to the generation of undesired bystander mutations by conventional CBE systems (Figure 1B).^[^ ^10^ ^]^ Before implementing CBE therapy in the mouse model, the primary challenge lies in screening the optimal CBE system to efficiently and precisely correct the *Pde6a* mutation. To address this challenge, we employed an adenine base editor (ABE) to generate a mouse Neuro‐2a (N2a) cell line carrying the *Pde6a* (c.2009A > G, p.D670G) mutation, identical to that in the *Pde6a* mouse model, which served as the N2a cell model (Figure S1, Supporting Information).^[^ ^11^ ^]^ Given that the target site of the *Pde6a* mutation lacks the canonical NGG protospacer‐adjacent motif (PAM) sequence, we designed two single guide RNAs (sgRNAs) with the NG PAM suitable for nCas9‐NG‐mediated CBEs (Figure 1B).^[^ ^12^ ^]^

To assess the efficacy of CBE editing, we transfected the CBE vectors with sgRNA1 or sgRNA2 into the N2a cells and determined the editing efficiency through sanger sequencing. Initially, we evaluated the classical rat APOBEC1 (rA1)‐mediated CBE systems (including rA1, rA1‐YE1, and rA1‐YEE)^[^ ^8^, ^13^ ^]^ in the N2a cell model (Figure S2A, Supporting Information). As anticipated, all rA1‐CBEs exhibited minimal editing at the target base (located in a GC context), while higher editing was observed at bystander 1 (located in a CC context), consistent with previous reports indicating that rA1 deaminase has a preference against the GC context^[^ ^14^ ^]^ (Figure 1C,D). Given the inefficiency of the rA1‐CBE system in repairing the *Pde6a* mutation, we subsequently tested other cytidine deaminase‐mediated CBE systems (including CDA1,^[^ ^15^ ^]^ engineered TadA,^[^ ^16^ ^]^ and AID^[^ ^17^ ^]^), which have been reported to possess better editing efficiency or context compatibility than rA1 (Figure S2B, Supporting Information). Among all tested CBE systems and sgRNAs, the combination of AID‐CBE and sgRNA1 demonstrated the highest efficiency in editing the target site, albeit with the highest bystander editing as well (Figure 1C,D). Most notably, we discovered that our previously designed AID‐N51G variant, which exhibits a slight preference for the GC context,^[^ ^14b^ ^]^ markedly reduced bystander mutations at this *Pde6a* locus (Figure 1C,D).

Encouraged by this observation, we pursued further design iterations of AID, aiming to develop an improved CBE system with enhanced efficiency and precision for correcting the *Pde6a* mutation. Based on previous structural studies of AID, we targeted five residues (R25, T27, N51, K52, and Y114) involved in base‐specific or non‐specific contacts to the deaminated cytidine in the catalytic pocket (Figure 1E).^[^ ^18^ ^]^ Among these, N51 is of particular interest as it is located near the active site and plays a critical role in deaminase activity and context preference.^[^ ^18^ ^]^ Based on this, we developed 11 engineered AID‐CBE variants with single amino acid substitutions and evaluated their performance in the N2a cell model. The results revealed that most of these AID variants maintain activity at the target base with reduced bystander mutations. Particularly noteworthy was the optimal AID‐N51A variant, which demonstrated significantly reduced bystander editing while maintaining high activity at the target base (Figure 1F). Furthermore, we investigated whether the precision of AID‐N51A could be enhanced by combining it with other point mutations (R25A or Y114F). However, this strategy evidently reduced target editing efficiency (Figure 1F). Given its superior efficiency in rescuing the *Pde6a* target mutation and relatively low bystander editing, we chose to proceed with the AID‐N51A‐CBE system paired with sgRNA1 for further in vivo CBE studies.

### 2.2 In Vivo Validation of *Pde6a* Mutation Correction by Electroporation of CBE Plasmids

**Figure 2:**
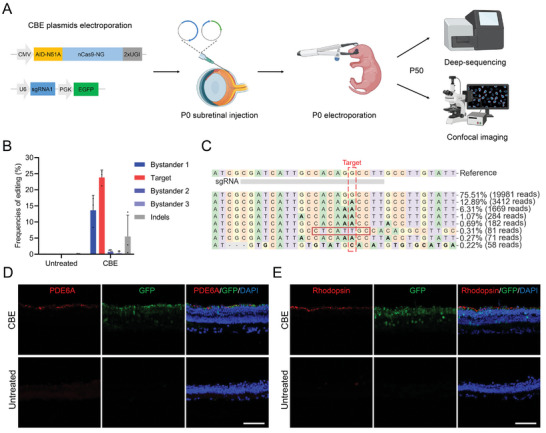
In vivo electroporation of CBE plasmids in *Pde6a* mice. A) Workflow for CBE subretinal injection of plasmids, electroporation, and rescue detection (Created by biorender.com). B) Editing efficiency of untreated and electroporated mouse retinas at the *Pde6a* site, as determined by deep sequencing (*n* = 3 eyes). C) Representative deep sequencing result of edited mouse retina at the *Pde6a* site. Target base, red dotted box; Substitutions, bold font; Insertions, red rectangles; Deletions, dashed lines. D,E) Representative immunofluorescence images of retinal sections examined with PDE6A (D) or Rhodopsin (E) antibodies in CBE‐electroporated or untreated *Pde6a* mice at P50. Nuclei were labeled with DAPI (blue). Scale bar, 50 µm.

In vivo electroporation is a well‐established method for the rapid and efficient delivery of DNA plasmids into the neonatal mouse retina.^[^ ^19^ ^]^ This technique has been extensively utilized to investigate retinal gene function and has even been applied in gene editing therapy studies.^[^ ^20^ ^]^ Here, we electroporated *Pde6a* mouse retinas with plasmids expressing optimal AID‐N51A‐CBE and sgRNA1 on postnatal day 0 (P0), utilizing co‐expressed EGFP as a marker of successful electroporation (**Figure** **2**A). Subsequently, the electroporated mouse retinas were dissected at P50, a timepoint at which the retina of *Pde6a* mice is nearly completely degenerated.^[^ ^4^, ^21^ ^]^ In order to analyze the rescue effect, deep sequencing and immunofluorescence (IF) were utilized (Figure 2A). The deep sequencing results revealed that the AID‐N51A‐CBE system induced an average correction efficiency of 23.8 ± 2.3% at the target base, demonstrating its effectiveness in vivo (Figure 2B,C). In addition to target editing, bystander editing was detected at bystander 1, with an average efficiency of 13.6 ± 4.7%, along with 5.5 ± 6.7% insertions and deletions (indels) (Figure 2B,C). No obvious off‐target editing was detected in the five predicted CBE off‐target sites (Figure S3, Supporting Information). A further result of the IF experiment indicated that the expression of both PDE6A and Rhodopsin, a marker of rod photoreceptors, had been restored following electroporation. (Figure 2D,E). In conclusion, these results suggested that the CBE system effectively repairs the *Pde6a* target mutation in vivo, albeit accompanied by a certain degree of bystander mutations.

### 2.3 In Vitro Screening for the Optimal PE System for *Pde6a* Mutation Correction

**Figure 3:**
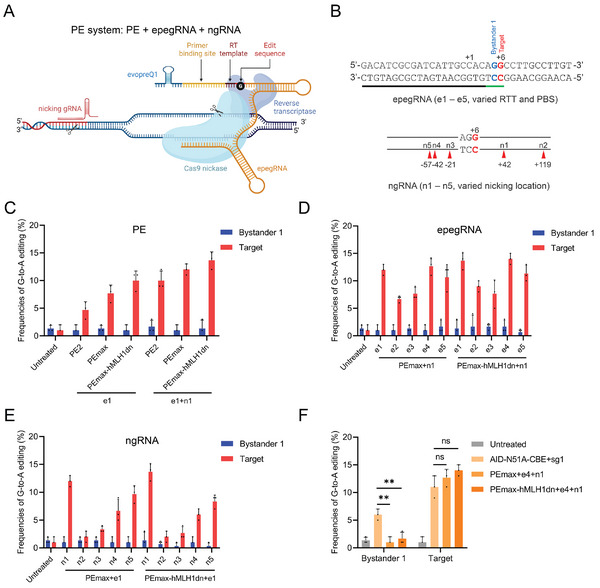
In vitro screening of the optimal PE system for *Pde6a* mutation correction. A) Illustration of the PE system (Created by biorender.com). Classical PE system consists of three components: the PE vector, epegRNA, and ngRNA. B) Schematic representation of epegRNAs and ngRNAs screening for *Pde6a* mutation correction. A total of 5 pegRNAs with varied lengths of RTT or PBS, and 5 ngRNA with varied nicking locations were evaluated. Target base, red; potential bystander base, blue; PAM sequence, green. C) Comparison of editing efficiencies among three different PE systems in N2a cells. (*n* = 3 biologically independent experiments). D) Comparison of editing efficiencies among five different epegRNAs in N2a cells. (*n* = 3 biologically independent experiments). E) Comparison of editing efficiencies among five different ngRNAs in N2a cells. (*n* = 3 biologically independent experiments). F) Comparison of target and bystander 1 editing efficiencies among optimal CBE and PE systems at the *Pde6a* site in N2a cells. (*n* = 3 biologically independent experiments). Two tailed unpaired t‐tests. **p < 0.01, ns: non‐significant difference.

The recently developed next‐generation gene editing tool, the PE system, is theoretically expected to possess higher precision than BE because it uses RT to write the target mutation into the genome rather than relying on DNA deamination of deaminases, which is prone to generating bystander mutations.^[^ ^7^, ^9^ ^]^ Therefore, we also explored the feasibility of using the latest PE system to correct *Pde6a* mutation (**Figure** **3**A). Following the PE experimental guidelines,^[^ ^22^ ^]^ we systematically designed and tested three PE vectors (PE2,^[^ ^9^ ^]^ PEmax and PEmax‐hMLH1dn^[^ ^23^ ^]^), along with five engineered PE guide RNAs^[^ ^24^ ^]^ (epegRNAs, e1 – e5) with recommended lengths of the primer binding site (PBS) and RT template (RTT) from PE design web tools (Figure 3B; Figure S4, Supporting Information).^[^ ^25^ ^]^ Moreover, we designed five nicking gRNAs (ngRNAs, n1 – n5) with a variety of non‐edited strand nicks, as previous reports suggested that nicks may enhance PE editing efficiency (Figure 3B).^[^ ^9^ ^]^ The test results in N2a cells indicated that the new PEmax and PEmax‐hMLH1dn vectors outperform the older PE2 vector, consistent with previous studies (Figure 3C).^[^ ^23^ ^]^ Among the five epegRNAs (e1 – e5), the e1 and e4 exhibited similar efficiency, surpassing the other three epegRNAs (Figure 3D). As for the five ngRNAs (n1 – n5), the n1 demonstrated the highest efficiency, significantly outperforming the other four ngRNAs (Figure 3E). In addition, recent reports have indicated that the introduction of silent mutations near the target edit can enhance PE editing efficiency by evading cellular mismatch repair (MMR).^[^ ^23^, ^26^ ^]^ However, the introduction of MMR‐evading mutations at this *Pde6a* locus did not significantly improve efficiency (Figure S5, Supporting Information). Finally, we selected the best‐performing combination of PEmax+e4+n1 or PEmax‐hMLH1dn+e4+n1 for comparison with the optimal AID‐N51A+sg1 CBE system in N2a cells (Figure 3F). We observed that the target editing efficiency of PE is comparable to that of CBE, while notably decreasing undesired mutations at bystander 1 (Figure 3F). Therefore, the in vitro testing of PE for correcting *Pde6a* mutation demonstrated exceptionally high precision, surpassing that of the CBE system.

### 2.4 In Vivo Validation of *Pde6a* Mutation Correction by PE Plasmids Electroporation

**Figure 4:**
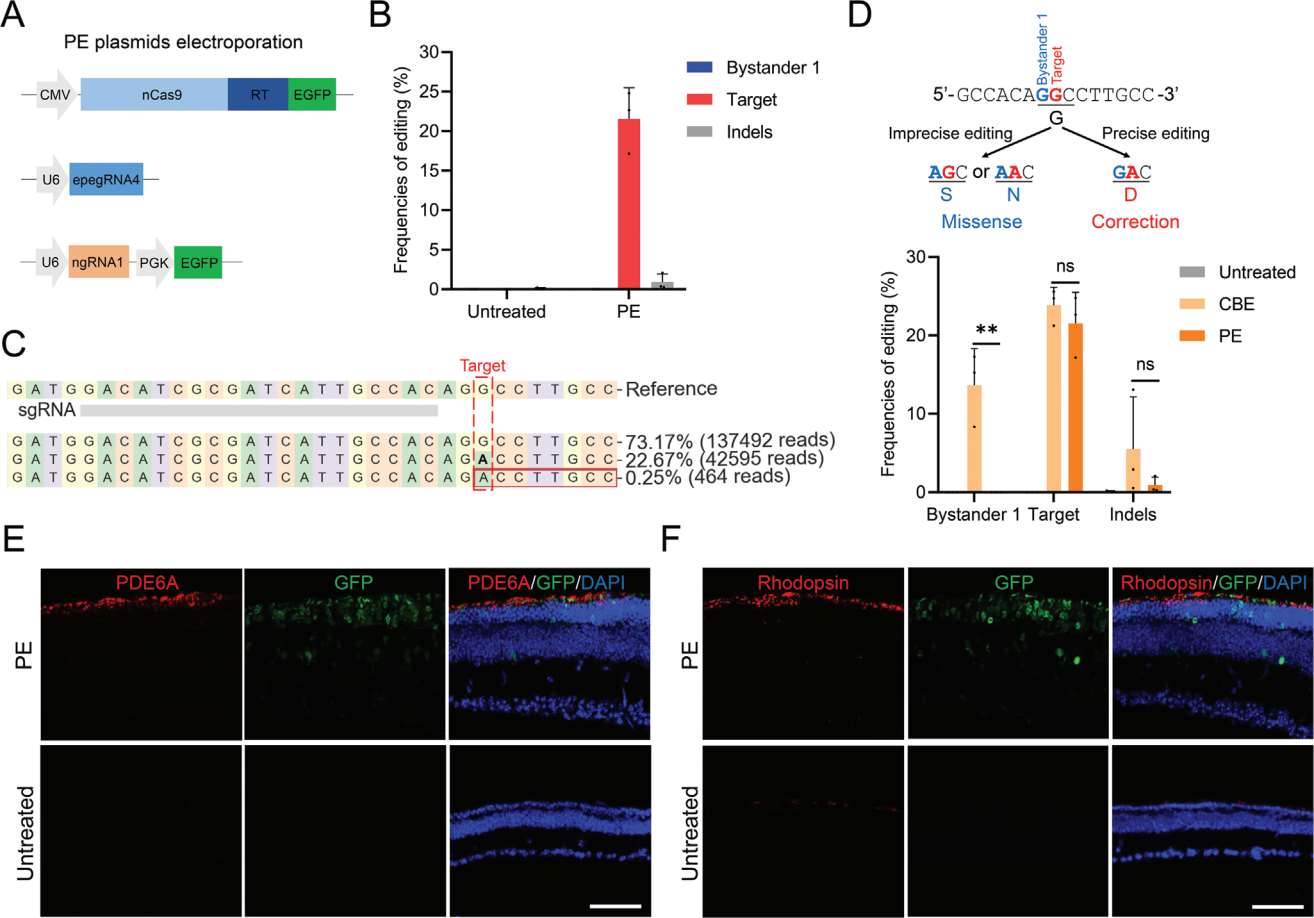
In vivo electroporation of PE plasmids in *Pde6a* mice. A) Schematic representation of electroporated PE plasmids. B) Editing efficiency of the untreated and electroporated mouse retina at the *Pde6a* site. (*n* = 3 eyes). C) Representative deep sequencing result of edited mouse retina at the *Pde6a* site. Target base, red dotted box; Substitutions, bold font; Insertions, red rectangles. D) Comparison of editing efficiencies between CBE‐ or PE‐electroporated mouse retinas at the *Pde6a* site. (*n* = 3 eyes). Two tailed unpaired t‐tests. **p < 0.01, ns: non‐significant difference. E,F) Representative immunofluorescence images of retinal sections examined with PDE6A (E) or Rhodopsin (F) antibodies in PE‐electroporated or untreated *Pde6a* mice at P50. Nuclei were labeled with DAPI (blue). Scale bar, 50 µm.

Encouraged by the precise PE correction observed in vitro, we proceeded to validate the in vivo efficiency through electroporation of PE plasmids. The three plasmids expressing the best‐performing PE systems, PEmax+e4+n1, were mixed and then electroporated into the retinas of newborn *Pde6a* mice (**Figure** **4**A). Electroporation of PE plasmids generated an average target editing efficiency of 21.5 ± 3.9% (*n* = 3), with no detectable bystander editing and 0.9 ± 1.0% indels (Figure 4B,C). Comparing the in vivo deep sequencing data of electroporated PE and CBE side by side, we found that PE exhibits similar target editing efficiency to CBE (21.5% versus 23.8%, *n* = 3) but eliminates unwanted bystander 1 editing (0% versus 13.6%, *n* = 3) (Figure 4D). Additionally, the frequencies of indels by PE are slightly lower than by CBE (0.9% versus 5.5%), although the difference is not significant (Figure 4D). Since bystander 1 and the target base jointly encode glycine (G670), theoretically, only precise target editing can achieve the correct rescue of G670D, while bystander1 editing caused by CBE will result in G670S or G670N, which still constitutes a missense mutation (Figure 4D). According to in situ fluorescent analysis of P50 mouse retinas, PE electroporation restored the expression of PDE6A and Rhodopsin, thereby ceasing the progressive degeneration of the retina (Figure 4E,F); rod photoreceptors were completely lost in the untreated group. In summary, these data demonstrated that the PE system corrects *Pde6a* mutation with higher precision than CBE, theoretically making it more suitable for in vivo therapeutic applications.

### 2.5 In Vivo PE Rescue of *Pde6a* Mutation by Dual AAV Delivery

**Figure 5:**
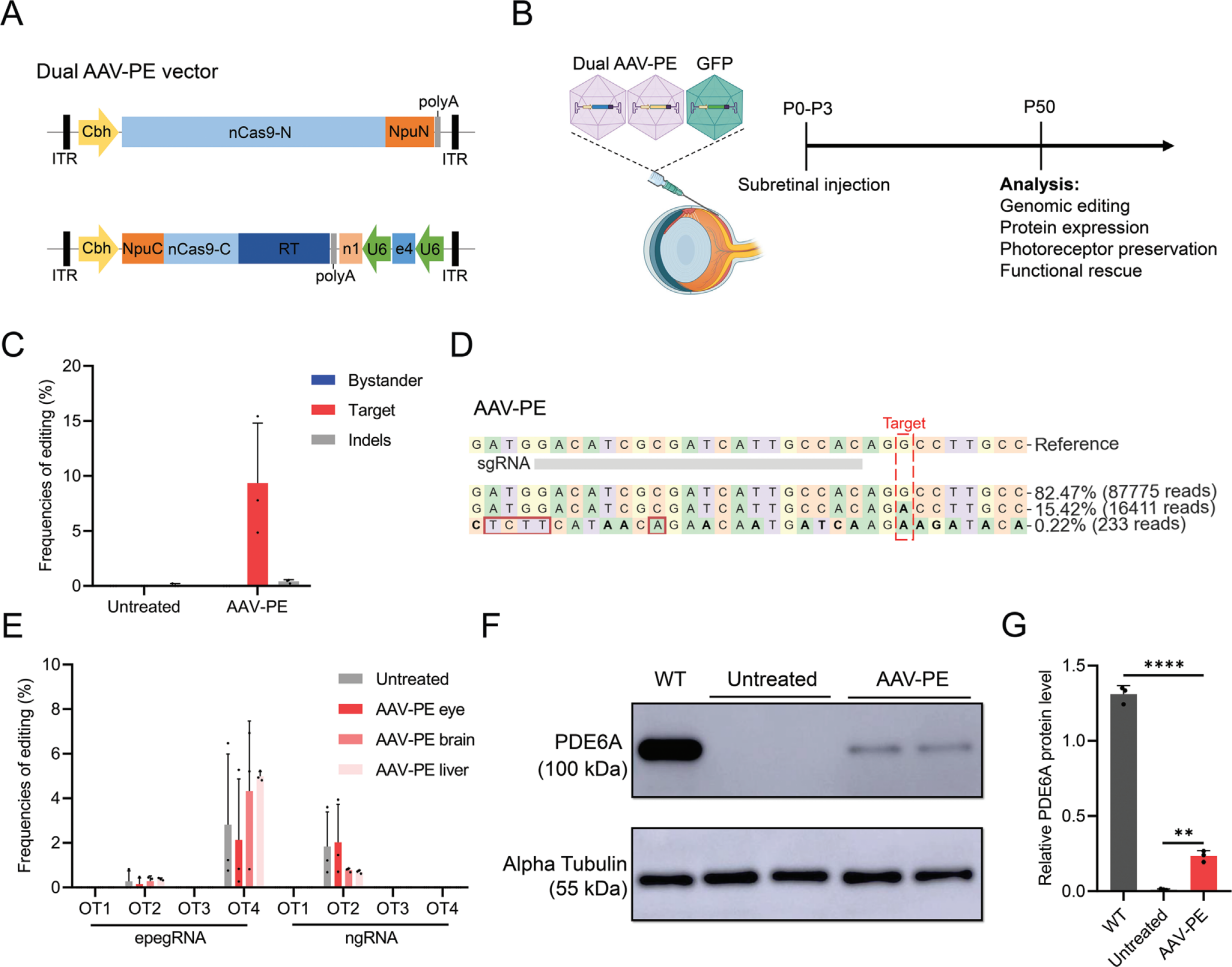
In vivo dual AAV‐PE treatment of the *Pde6a* mutation. A) Schematic representation of constructed dual AAV‐PE vectors for AAV delivery. B) Experimental outline of the AAV injection and subsequent in vivo analyses in *Pde6a* mice. C) In vivo editing efficiency in *Pde6a* mice after AAV‐PE treatment (*n* = 3 eyes). D) Representative deep sequencing result of AAV‐PE treated mouse retina at the *Pde6a* site. Target base, red dotted box; Substitutions, bold font; Insertions, red rectangles. E) Off‐target editing efficiencies in *Pde6a* mice after AAV‐PE treatment (*n* = 3 eyes). OT, off‐target. F) Western blot analysis of PDE6A protein expression in WT mice, untreated and AAV‐PE treated *Pde6a* mice at P50. G) Quantification of the relative PDE6A protein level normalized to Alpha tubulin. (*n* = 3 repeated experiments). One‐way ANOVA with Tukey's multiple comparison tests. **p < 0.01, ****p < 0.0001.

While electroporation of PE plasmids can effectively correct the target mutation in *Pde6a* mice, this method cannot be applied to translational treatment in humans. The AAV, as a mature gene therapy delivery platform, has been widely used to deliver cDNA or gene editing components for clinical or preclinical experiments in the treatment of eye diseases.^[^ ^27^ ^]^ Considering the limitation of AAV package capacity, we utilized a split Npu intein mediated dual‐AAV system,^[^ ^28^ ^]^ where one AAV vector expresses the N‐terminal portion of PE, and the other AAV vector expresses the C‐terminal portion, along with epegRNA4 and ngRNA1 (**Figure** **5**A). Due to the presence of split Npu intein, the two halves of the PE would splice in trans and reconstitute a whole PE complex in vivo.^[^ ^28^ ^]^ The dual AAV‐PE vector demonstrated editing efficiency like that of the intact PE vector in N2a cells (Figure S6, Supporting Information). Subsequently, we employed the AAV2.NN serotype, a derivative of AAV2 with improved retinal transduction properties,^[^ ^29^ ^]^ to deliver the dual AAV‐PE system to *Pde6a* mice by subretinal injection at P0‐P3 (Figure 5B). A small amount of AAV‐GFP was co‐delivered to confirm successful AAV transduction. The retinas were subsequently harvested and assayed at P50 when retinal degeneration is nearly complete (Figure 5B). Deep sequencing of the genomic DNA extracted from the retina tissues of the treated mice revealed a 9.4 ± 5.5% correction of the target mutation, with 0.4 ± 0.2% indels (Figure 5C,D). No bystander mutations were detected, further confirming the high precision of the PE system in rescuing the *Pde6a* mutation (Figure 5C,D). We examined the eight predicted potential off‐target sites to determine off‐target effects in the eyes and found no obvious off‐target editing above the background level of the untreated group (Figure 5E). In addition to the eyes, we also evaluated off‐target editing in two non‐targeted organs, the brain and liver, and found no significant off‐target editing, further demonstrating the safety of AAV‐PE (Figure 5E). It was found that PDE6A protein expression was partially restored in treated mice, though it was at a weaker level than in wild‐type mice (Figure 5F,G). However, no PDE6A protein was detected in the eyes of untreated mice. Taken together, these findings suggested that our dual AAV‐PE system precisely corrected the pathogenic *Pde6a* mutation in vivo without any bystander editing and restored PDE6A expression.

### 2.6 Photoreceptor Preservation in *Pde6a* Mice by AAV‐PE Treatment

**Figure 6:**
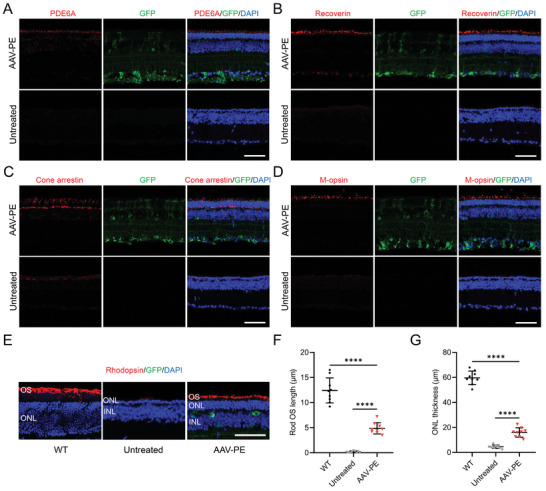
Preservation of retinal photoreceptors in *Pde6a* mice by AAV‐PE treatment. A–D) Representative immunofluorescence images of retinal sections examined with PDE6A (A), Recoverin (B), Cone arrestin (C) or M‐opsin (D) antibodies in untreated and AAV‐PE treated *Pde6a* mice at P50. Nuclei were labeled with DAPI (blue). Scale bar, 50 µm. E) Immunofluorescence analysis of representative retinal sections examined for Rhodopsin. OS, outer segments; ONL, outer nuclear layer; INL, inner nuclear layer. Scale bar, 50 µm. F) Quantification of rod OS length in WT mice, untreated and AAV‐PE treated *Pde6a* mice at P50 (*n* = 3 eyes, three values per eye). One‐way ANOVA with Tukey's multiple comparison tests. ****p < 0.0001. G) Quantification of ONL thickness in WT mice, untreated and AAV‐PE treated *Pde6a* mice at P50 (*n* = 3 eyes, three values per eye). One‐way ANOVA with Tukey's multiple comparison tests. ****p < 0.0001.

Retinal photoreceptors, including rod photoreceptors and cone photoreceptors, gradually die during the process of retinal degeneration in *Pde6a* mice.^[^ ^4^ ^]^ We examined retinal sections with IF to determine whether the correction of the Pde6a mutation preserved retinal photoreceptors. Retinas from P50 mice were cryosectioned and immunostained with antibodies against PDE6A, a rod‐specific marker (Recoverin), and cone‐specific markers (Cone arrestin and M‐opsin). The AAV‐PE treated retinas showed prominent PDE6A immunolabeling consistent with the western blot results (**Figure** **6**A; Figure S7, Supporting Information). In comparison to the weak or nearly undetectable signals of Recoverin, Cone arrestin, and M‐opsin in untreated retinas, these phototransduction‐relevant proteins exhibited robust expression and appropriate localization in retinas treated with AAV‐PE, indicating remarkable preservation of rods and cones (Figure 6B–D; Figure S7, Supporting Information). In addition, we immunolabeled retinal sections with Rhodopsin antibody to visualize and measure the rod outer segments (OS) (Figure 6E). In AAV‐PE treated retinas, the rod OS were clearly visible, with a length of 4.9 ± 1.1 µm, ≈40% of the length observed in WT retinas (12.4 ± 2.5 µm), whereas the OS were almost invisible in untreated retinas (Figure 6E,F). In addition, the untreated retinas exhibited only 1–2 layers of nuclei in the outer nuclear layer (ONL) at P50, whereas the AAV‐PE treated retinas displayed a significantly thicker ONL (Figure 6E). The quantitative assay of retinal sections revealed that ONL thickness measured 15.9 ± 3.8 µm in the AAV‐PE group, representing 3.5‐fold increases compared to the untreated group (Figure 6G). In addition, the HE staining data further confirmed the similar results (Figure S8, Supporting Information). Overall, these results strongly indicated that AAV‐PE treatment effectively preserves the photoreceptor morphology in *Pde6a* mice.

### 2.7 Rescue of Retinal Function in *Pde6a* Mice by AAV‐PE Treatment

**Figure 7:**
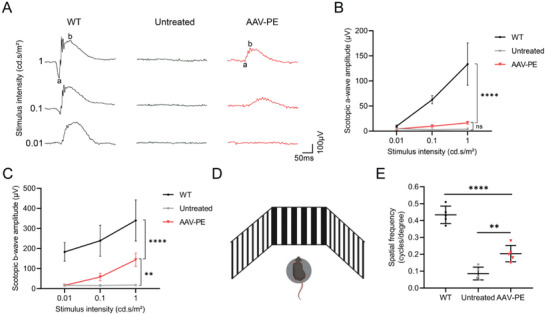
Rescue of retinal function in *Pde6a* mice by AAV‐PE treatment. A) Representative scotopic ERG response of WT mice, untreated and AAV‐PE treated *Pde6a* mice at P50. B) Quantification of scotopic ERG a‐wave amplitudes from each group at P50 (*n* = 5 mice). Two‐way ANOVA with Tukey's multiple comparison tests. ****p < 0.0001, ns: non‐significant difference. The source data were provided in Table S4 (Supporting Information). C) Quantification of scotopic ERG b‐wave amplitudes from each group at P50 (*n* = 5 mice). Two‐way ANOVA with Tukey's multiple comparison tests. **p < 0.01, ****p < 0.0001. The source data were provided in Table S4 (Supporting Information). D) Schematic of optokinetic tracking response (OKR) test in mice. E) Quantification of visual acuity in WT mice, untreated and AAV‐PE treated *Pde6a* mice at P50 by OKR testing (*n* = 5 mice). One‐way ANOVA with Tukey's multiple comparison tests. **p < 0.01, ****p < 0.0001.

Previous reports have indicated that this *Pde6a* RP mouse model exhibits severe photoreceptor degeneration and impaired retinal function.^[^ ^4^, ^30^ ^]^ To assess whether the morphological preservation of the retina maintained visual function, we used electroretinography (ERG) to measure the electrical activity of photoreceptors at P50. We recorded scotopic ERG signals in dark‐adapted WT, untreated and AAV‐PE treated mice using a series of light stimulus intensity increasing from 0.01 to 1 cd s⁻^1^ m⁻^2^. As shown in **Figure** **7**A, the representative ERG traces of the AAV‐PE treated mice showed moderate rescue of visual function in response to the stimuli compared to the untreated mice. Quantitative analysis indicated a significant increase in both a‐wave and b‐wave amplitudes in AAV‐PE treated mice compared to untreated mice although they were weaker than those of WT mice (Figure 7B,C). At a stimulus intensity of 1 cd s⁻^1^ m⁻^2^, the AAV‐PE treated mice exhibited a‐wave and b‐wave amplitudes that were ≈12% and 42%, respectively, of the ERG amplitudes from WT mice (a‐wave: 16.5 ± 3.0 µV vs 133.6 ± 42.1 µV; b‐wave: 143.8 ± 33.7 µV vs 339.8 ± 102.6 µV). In contrast, ERG signals were almost undetectable in untreated mice (Figure 7B,C). To assess the impact of AAV‐PE treatment on visual acuity, we also measured optokinetic tracking response (OKR) to quantify visual acuity in P50 mice (Figure 7D). AAV‐PE treated mice also exhibited higher visual acuity than untreated mice in the OKR test (Figure 7E). Together, these results demonstrated that in vivo application of AAV‐PE can partially rescue retinal function in *Pde6a* mice.

Additionally, we also attempted AAV‐PE injection at P14, a time point when *Pde6a* mice have already begun to exhibit retinal degeneration, which better simulates translational treatment conditions.^[^ ^4^ ^]^ Our results showed that the P14 injection also exhibited obvious therapeutic effect, though it was slightly less effective than the injection at P0 (Figure S9, Supporting Information).

## 3 Discussion

CRISPR‐based BE and PE techniques, capable of inducing precise and efficient single‐base modifications, hold immense potential for the permanent and effective treatment of numerous genetic disorders, especially inherited retinal conditions.^[^ ^7^, ^27^ ^]^ The eye presents a particularly advantageous target for gene therapy because its relatively autonomous and immune‐privileged status facilitates straightforward administration, delivery, and monitoring of therapeutic outcomes.^[^ ^27b^ ^]^ In this study, we systematically demonstrated the effectiveness of both engineered CBE and PE systems in correcting the RP related *Pde6a* (c.2009A>G, p.D670G) mutation both in vitro and in vivo. Crucially, the dual AAV‐delivered PE system effectively and precisely corrected the target mutation, restored PDE6A expression, preserved photoreceptors, and rescued retinal function in *Pde6a* mice, thereby demonstrating its potential in treating RP.

The traditional AAV‐*Pde6a* gene supplementation method has shown promising rescue in animal models,^[^ ^5^, ^31^ ^]^ but limited success in the clinical treatment of *Pde6a* patients.^[^ ^6^ ^]^ In studies using the same *Pde6a*‐D670G mouse model, AAV‐*Pde6a* cDNA treatment significantly delayed retinal degeneration, preserving ONL thickness and restoring ERG responses, with effects comparable to or even surpassing those of our AAV‐PE treatment.^[^ ^5^ ^]^ In comparison, AAV‐PE treatment directly repairs genomic DNA mutations, restoring the expression of endogenous PDE6A protein and potentially addressing concerns about the declining transgene expression over time.^[^ ^27^, ^32^ ^]^ However, further studies are needed to confirm this hypothesis. Additionally, the editing efficiency of AAV‐PE needs to be further improved to enhance its therapeutic effectiveness to a level comparable with AAV‐*Pde6a* cDNA. Dual AAV‐PE delivery may present more disadvantages compared to AAV‐cDNA, including instability, complexity in delivery, the need for higher doses, increased immunogenicity, and a greater risk of off‐target effects. These issues need to be evaluated in future studies.

The BE and PE require the design of different sgRNAs or epegRNAs for each specific mutation, which is less straightforward and universal than the gene supplementation method. Although we have identified the most optimal BE or PE systems and corresponding gRNAs for the *Pde6a* (c.2009A>G, p.D670G) mutation and conducted preliminary validation of the therapeutic effect in a mouse model, further evaluations of efficiency and safety are required to advance to translational applications. Additionally, treating other clinically relevant point mutations in the *Pde6a* gene will still require screening for the optimal BE or PE system for each mutation, which demands additional time and financial investment. This remains a major challenge for the translational application of BE and PE therapies.

In addition, the CBE and PE systems currently are too large to fit within the ≈4.7‐kb cargo size limit of AAV vectors. The current mainstream approach is to use the dual AAV‐delivered split CBE or PE to bypass this limitation, but this may decrease the delivery and editing efficiency.^[^ ^28^, ^33^ ^]^ A potential solution is to utilize non‐viral delivery methods, such as nanoparticles, episomal vectors or virus‐like particles (VLPs), thereby bypassing the need for AAV vectors.^[^ ^7^, ^34^ ^]^ Another alternative strategy would be to use compact CRISPR or transposon‐encoded RNA‐guided nucleases, like Un1Cas12f1 (529 aa),^[^ ^35^ ^]^ TnpB (≈400 aa),^[^ ^36^ ^]^ or IscB (≈500 aa).^[^ ^37^ ^]^ These smaller nucleases, approximately half the size of current SpCas9, could potentially replace Cas9 in the BE or PE system, allowing for a reduction in size for single AAV delivery. However, further investigation is needed to assess their efficiency.

## 4 Experimental Section

### Animals

Wild‐type C57BL/6 mice were purchased from the Charles River Laboratories (#027). *Pde6a* ^nmf363/nmf363^ mice^[^ ^4^ ^]^ were a gift from Dr. Vinit B. Mahajan (Stanford Ophthalmology). Animals were housed under a 12‐h light/12‐h dark cycle with access to water and food. All animal experimental procedures were performed in compliance with animal protocol (#32223) approved by the IACUC at Stanford University School of Medicine.

### Plasmid Construction

All CBE plasmids used in this study were based on the BE4max‐NG backbone (CMV‐deaminase‐(nCas9‐NG)−2xUGI).^[^ ^38^ ^]^ The rA1‐NG‐CBE (#125617), rA1‐YE1‐NG‐CBE (#138159), CDA1‐NG‐CBE (#125612), AID‐CBE (#174696), and ABE8e‐nSpRY (#185671) plasmids were obtained from Addgene. The rA1‐YEE‐NG, TadA‐CBE, TadA‐N46L‐CBE, AID‐NG‐CBE, and AID variants‐NG‐CBE were constructed by site directed‐mutagenesis and DNA recombinant cloning using Fast Site‐Directed Mutagenesis Kit (Vazyme, #C214) and ClonExpress Ultra One Step Cloning Kit (Vazyme, #C115). For PE, the PE2 (#132775), PEmax (#180020), PEmax‐hMLH1dn (#174828), dual AAV‐PE‐N (#198734) and dual AAV‐PE‐C (#198735) plasmids were obtained from Addgene. The epegRNA plasmids were constructed using the pU6‐tevopreq1‐GG‐acceptor (#174038), following published protocols.^[^ ^9^, ^22^ ^]^ The ABE‐sgRNA, CBE‐sgRNA or PE‐ngRNA were generated by T4 ligation of annealed oligos into the *BsaI*‐digested U6‐gRNA‐PGK‐Puro (#51133) or U6‐gRNA‐PGK‐EGFP (#107721) plasmid. The sequences of gRNAs are listed in Table S1 (Supporting Information).

### Cell Line Generation

The N2a cells were transfected with ABE8e‐nSpRY and U6‐gRNA‐PGK‐Puro plasmids to install the *Pde6a* (c.2009A>G, p.D670G) mutation, identical to the mutation found in *Pde6a* ^nmf363/nmf363^ mice. Transfected cells were then subjected to puromycin selection at a concentration of 2 µg mL⁻^1^ for 5 days. Subsequently, the cells were diluted and plated onto 96‐well plates for amplification and culture. Single cell colonies were chosen for sanger sequencing verification. Finally, the N2a single cell colony carrying the correct *Pde6a* (c.2009A > G, p.D670G) mutation was expanded to serve as the N2a cell model for CBE or PE testing. The sgRNA used for constructing the mutant cell line is listed in Table S1 (Supporting Information).

### Cell Culture and Transfection

The N2a cell line was cultured in Dulbecco's Modified Eagle's Medium (Corning, #10013CV) supplemented with 10% fetal bovine serum and incubated at 37 °C in an atmosphere of 5% CO_2_. The cells were seeded in 24‐well plates and transfected using PolyJet In Vitro DNA Transfection Reagent (SignaGen Laboratories, #SL100688) according to the manufacturer's instructions. Briefly, 1.5 µL of PolyJet reagent with total 500 ng BE or PE plasmids were added to each well. After 72 h, the transfected cells were lysed by One Step Mouse Genotyping Kit (Vazyme, #PD101) according to the manufacturer's instructions. The primers used to amplify target sequences are listed in Table S2 (Supporting Information). Sanger sequencing results were analyzed by EditR to determine the BE efficiency.^[^ ^39^ ^]^

### In Vivo Electroporation

The in vivo mouse retina electroporation was carried out with previously described methods.^[^ ^19^, ^20^ ^]^ Briefly, newborn P0 *Pde6a* mice were anesthetized by chilling on ice, and their eyelids were carefully opened with a sharp 30‐gauge needle. High concentrations of BE or PE plasmids were extracted using a PureLink HiPure Plasmid Midiprep Kit (Thermo Fisher Scientific, #K210004), and their concentrations were measured with a Thermo Scientific NanoDrop Spectrophotometer, typically exceeding 3000 ng µL⁻^1^. Before injection, these plasmids were diluted in 1xPBS as needed to ensure a final concentration of 1000 ng µL⁻^1^ for each plasmid. The ≈0.3–0.5 µL CBE or PE DNA solutions (1000 ng µL⁻^1^ of each plasmid, with 0.1% FastGreen dye as an injection tracer) were injected into the subretinal space by FemtoJet 4i microinjector (Eppendorf, #5252000021); the head of the injected pup was placed between a 10 mm diameter tweezer electrode and five pulses of 80 V, 50 ms each were applied at an interval of 950 ms by an NEPA21 electroporator (Bulldog Bio).

### AAV Production and Injection

The dual AAV‐PE was packaged with serotype AAV2.NN^[^ ^29a^ ^]^ and generated by the AAVnerGene. The titer of the produced AAV was 2 × 10^13^ GC mL⁻^1^. For AAV delivery, *Pde6a* mice received ≈0.5 µL AAV (the ratio of AAV‐PE‐N and AAV‐PE‐C is 1:1) with a small amount of AAV‐GFP (≈0.1 µL) as indicator, per eye via subretinal injection at P0‐P3. Mice were anesthetized on ice and subretinal injections were administered using a custom‐crafted glass micropipette and FemtoJet 4i microinjector (Eppendorf, #5252000021). For P14 injection, mice were anesthetized by ketamine, and pupils were dilated by 1% topical tropicamide. Subretinal injections were administered under an ophthalmic surgical microscope with Picospritzer III microinjection system and a custom‐crafted glass micropipette. ≈0.5 µL AAV was injected into the subretinal space through a small scleral incision.

### Targeted Deep DNA Sequencing

Genomic DNA was extracted from electroporated or AAV treated GFP regions of mouse retinas using FastPure Cell/Tissue DNA Isolation Mini Kit (Vazyme, #DC102) according to the manufacturer's protocol. The potential off‐target sites for CBE‐sgRNA, PE‐epegRNA, and PE‐ngRNA were predicted using Cas‐OFFinder.^[^ ^40^ ^]^ Deep sequencing primers were designed with generic adapters, and PCR was performed using Phusion High‐Fidelity DNA Polymerase (Thermo Scientific, #F530L). Targeted deep DNA sequencing was conducted by Amplicon‐EZ sequencing service in Azenta Life Sciences. Data analysis was performed with CRISPResso2.^[^ ^41^ ^]^ The primers used to amplify on‐target and off‐target sequences are listed in Tables S2 and S3 (Supporting Information).

### Western Blot Analysis

For western blot, the mouse retinas were dissected and homogenized in 200 uL of RIPA Lysis Buffer (Millipore Sigma, #20‐188) supplemented with a protease inhibitor cocktail (Thermo Scientific, #78430). The protein concentrations were measured by the Pierce BCA Protein Assay Kit (Thermo Scientific, #23227). Anti‐PDE6A antibody (Novus Biologicals, #NBP1‐87312, 1:500) and anti‐Alpha Tubulin antibody (Proteintech, #11224‐1‐AP, 1:5000) were used as primary antibody and internal control, respectively. Signals were acquired by direct measurement of chemiluminescence using a digital camera (Amersham Imager 600).

### Immunofluorescence Analysis

Mice were euthanized using CO_2_, and eyeballs were enucleated and fixed in 4% PFA. Retinas were carefully dissected, subjected to a sucrose gradient series (5%, 15%, 30% sucrose), embedded in OCT compound, and stored at −80 °C. Cryosections of 15 mm thickness were prepared using a Leica CM1950 cryostat (Leica Biosystems). The retinal cryosections were rinsed in PBS, blocked in a solution of 0.1% Triton X‐100 and 3% BSA in PBS for 30 min at room temperature, and then incubated overnight at 4 °C with primary antibodies diluted in the blocking buffer within a humidified chamber. Following three PBS washes with 0.1% Triton, sections were exposed to secondary antibodies for 2 h. DAPI was used to counterstain cell nuclei for 10 min. Slides were then mounted using Fluoromount‐G mounting medium (Southern Biotech) and covered with a coverslip. The following antibodies were used: rabbit anti‐PDE6A (Novus Biologicals, #NBP1‐87312, 1:500), mouse anti‐Rhodopsin (Abcam, #ab5417, 1:1000), rabbit anti‐Recoverin (Millipore, #AB5585‐I, 1:500), rabbit anti‐M‐opsin (Millipore, #AB5405, 1:500) and rabbit anti‐Cone arrestin (Millipore, #AB15282, 1:500). The Alexa‐Fluor‐555‐conjugated anti‐mouse IgG (Invitrogen, # A‐21147, 1:500) or anti‐rabbit IgG (Invitrogen, #A21428, 1:500) was used as secondary antibody. All images of retinal sections were captured by a Zeiss LSM880 inverted confocal microscope.

### H&E Staining Analysis

Following the standard H&E staining protocol, the slides with retinal sections were stained with H&E and mounted with Fisher Chemical Permount Mounting Medium (Fisher Scientific, #SP15‐100). Images were captured with a Keyence BZ‐X800 Microscope.

### Electroretinography (ERG)

Mice dark‐adapted for 12 h before ERG recording were anesthetized by ketamine based on their body weight (0.08 mg ketamine/g + 0.01 mg xylazine), and their pupils were dilated by 1% tropicamide. The ERG was performed with an ERG stimulator (Celeris, Diagnosys LLC) according to the manufacturer's instructions. Mice were stimulated with flashes of 0.01, 0.1, and 1 cd s⁻^1^ m⁻^2^ light intensity.

### Optokinetic Tracking Response (OKR)

The detailed procedure has been previously published.^[^ ^42^ ^]^ Briefly, the OKR was assessed using the OptoMotry system (CerebralMechanics Inc.), a virtual‐reality platform designed to swiftly quantify visuomotor behavior. Mice were positioned on a central platform surrounded by four computer monitors equipped with a video camera positioned overhead to record the animal's movements. A rotating cylinder displaying vertical sine‐wave gratings was projected onto the monitors. The OptoMotry software controlled the spatial frequency of the grating to assess the spatial acuity (cycle/degree) of the mouse being tested. The mouse's tracking of the gratings was reflected through head and neck movements. The maximum spatial frequency of each eye was determined by gradually increasing the spatial frequency of the grating until the mouse ceased tracking.

### Statistical Analysis

All data are expressed as mean ± SD of at least three individual determinations for all experiments. Statistical analysis was performed via GraphPad prism software 8.0.1. The statistical tests used for each experiment are stated in the corresponding figure legends.

## 5 Conclusion

In summary, we utilized engineered CBE and PE systems that induced efficient *Pde6a* mutation correction in vitro and in vivo. The PE system showed comparable target editing efficiency to CBE but eliminated undesirable bystander mutations, rendering it more suitable for therapeutic use. Treatment of *Pde6a* mice with dual AAV‐PE partly restored retinal photoreceptor morphology and visual function. Therefore, these engineered CBE and PE systems present promising therapeutic avenues for the treatment of RP.

## Conflict of Interest

The authors declare no conflict of interest.

## Supporting information



Supporting Information

## Data Availability

The data that support the findings of this study are available in the Supporting Information of this article.
