# Supplementary material for: Efficient Rescue of Retinal Degeneration in Pde6a Mice by Engineered Base Editing and Prime Editing
Source: Adv Sci (Weinh). 2024 Sep 19;11(42):2405628. doi: 10.1002/advs.202405628 (PMC11558111; doi:10.1002/advs.202405628)

ADVANCED SCIENCE Open Access

Supporting Information

for *Adv. Sci.*, DOI 10.1002/advs.202405628

Efficient Rescue of Retinal Degeneration in *Pde6a* Mice by Engineered Base Editing and Prime Editing

*Zhiquan Liu, Siyu Chen, Alexander E. Davis, Chien-Hui Lo, Qing Wang, Tingting Li, Ke Ning, Qi Zhang, Jingyu Zhao, Sui Wang and Yang Sun**

Supporting Information

Efficient Rescue of Retinal Degeneration in *Pde6a* Mice by Engineered Base Editing and Prime Editing

*Zhiquan Liu, Siyu Chen, Alexander E. Davis, Chien-Hui Lo, Qing Wang, Tingting Li, Ke Ning, Qi Zhang, Jingyu Zhao, Sui Wang, Yang Sun**

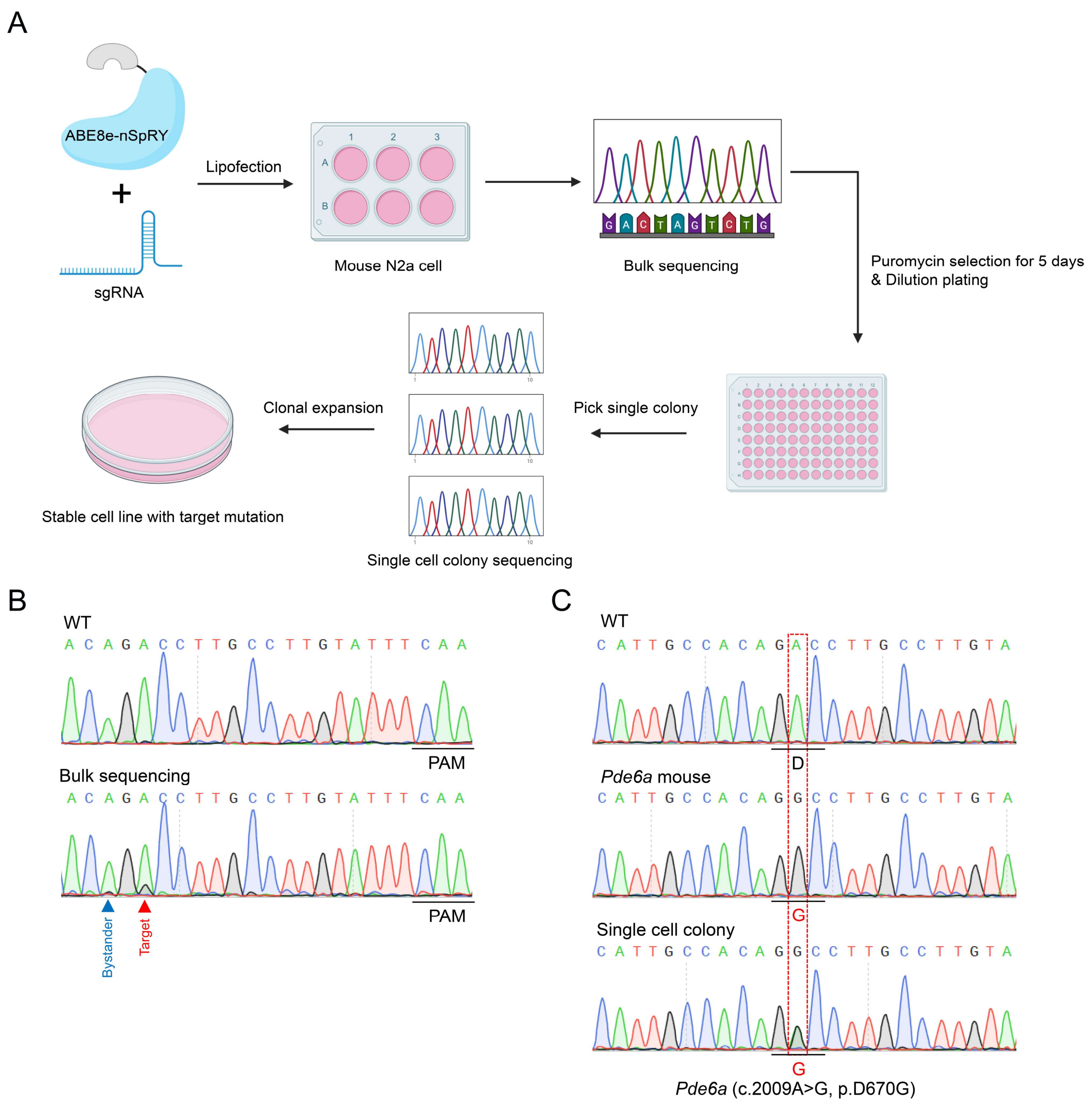

Supplementary Figure S1. Generation of a N2a cell line carrying the *Pde6a* (c.2009A>G, p.D670G) mutation. **A** Experimental pipeline of constructing the N2a cell line stably carrying the *Pde6a* mutation. **B** Sanger sequencing chromatograms of edited bulk N2a cells at the *Pde6a* site. Target editing, red; Bystander editing, blue. **C** Sanger sequencing chromatograms of edited single cell colony with precise heterozygous A-to-G mutation at the *Pde6a* site. Other obtained single cell colonies carried bystander editing and thus were not utilized (Data not shown).

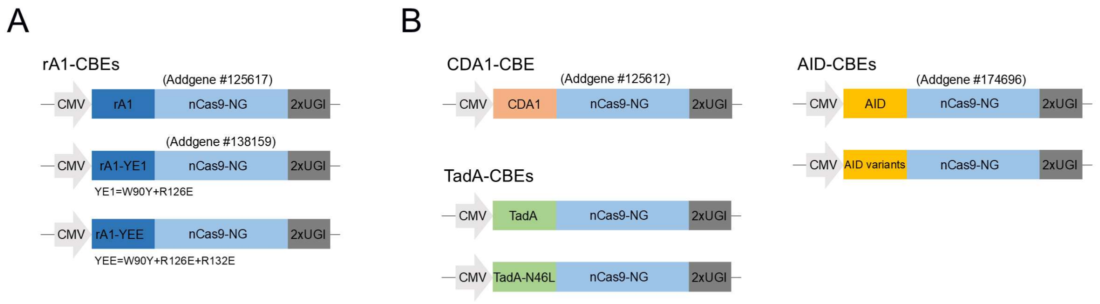

Supplementary Figure S2. Schematic representation of constructed CBE vectors of rA1-CBEs, CDA1-CBE, TadA-CBEs and AID-CBEs.

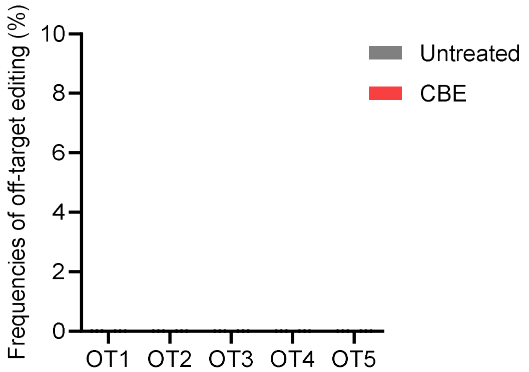

Supplementary Figure S3. Off-target editing efficiencies of untreated and CBE electroporated mouse retinas, as determined by deep sequencing (n=3 eyes).

A

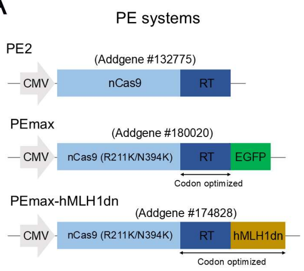

B

	PBS length	PBS sequence	RTT length	RTT sequence	Web tool
epegRNA1	13	GCAATGATCGCGA	14	AGGCAAGGTCTGTG	pegFinder (1)
epegRNA2	15	GCAATGATCGCGATG	17	ACAAGGCAAGGTCTGTG	Prime Design (2)
epegRNA3	13	GCAATGATCGCGA	17	ACAAGGCAAGGTCTGTG	pegIT (3)
epegRNA4	12	GCAATGATCGCG	13	GGCAAGGTCTGTG	Easy-Prime (4)<br>or DeepPrime (5)
epegRNA5	15	GCAATGATCGCGATG	10	AAGGTCTGTG	PRIDICT (6)

Supplementary Figure S4. A Schematic representation of three used PE vectors. The conventional PE2 utilized nCas9 (H840A) fused with MMLV reverse transcriptase (RT). PEmax has undergone numerous optimizations compared to PE2, primarily including the introduction of two additional mutations in nCas9 (R211K/N394K) and further codon optimization. PEmax-hMLH1dn incorporated a human dominant negative MMR protein (hMLH1dn) to inhibit cellular MMR based on the PEmax. **B** The detailed parameters of used epegRNAs in this study. They were sourced from the recommendations of various PE design web tools. PBS, primer binding site; RTT, reverse transcriptase template.

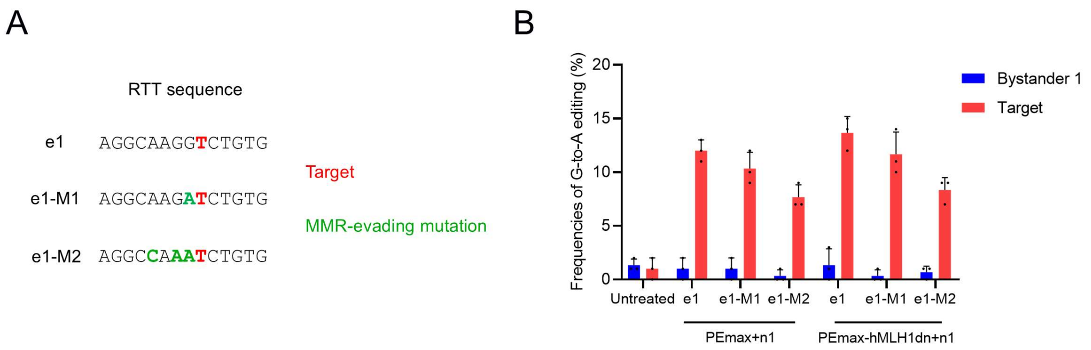

Supplementary Figure S5. A Schematic representation of designed epegRNAs with MMR-evading mutations. **B** Comparison of editing efficiencies among epegRNAs with MMR-evading mutations in N2a cells. (n=3 biologically independent experiments).

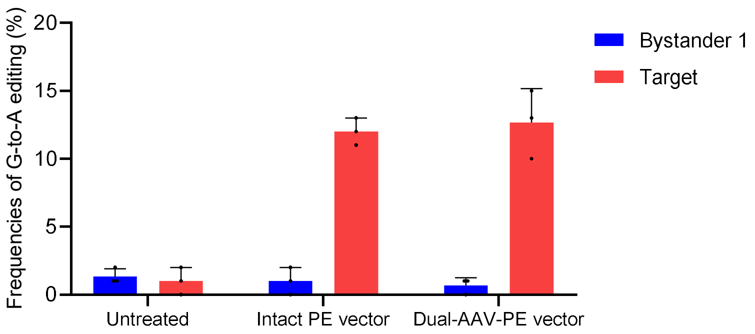

Supplementary Figure S6. Comparison of editing efficiencies between the intact PE vector and the dual AAV-PE vector at *Pde6a* site in N2a cells. (n=3 biologically independent experiments).

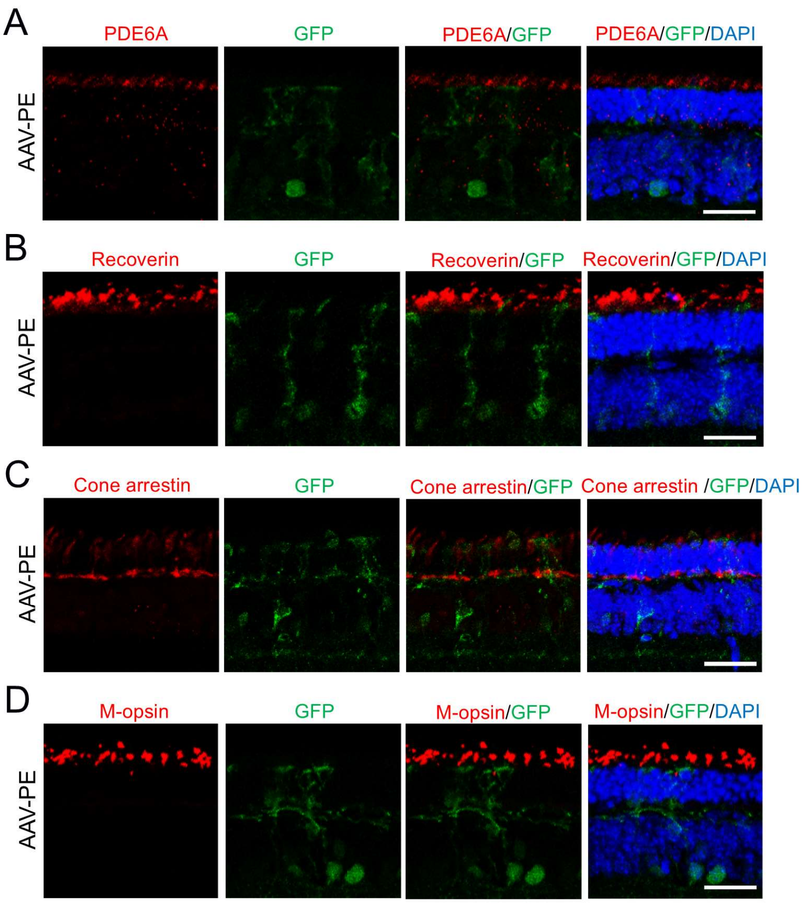

Supplementary Figure S7. The high magnification images from Figure 6. **A-D** Representative immunofluorescence images of retinal sections examined with PDE6A (**A**), Recoverin (**B**), Cone arrestin (**C**) or M-opsin (**D**) antibodies in untreated and AAV-PE treated *Pde6a* mice at P50. Nuclei were labelled with DAPI (blue). Scale bar, 25 μm.

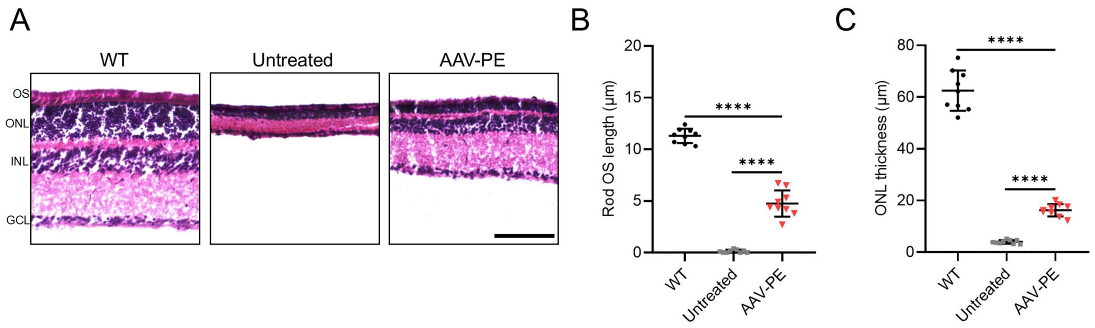

Supplementary Figure S8. A H&E staining analysis of representative retinal sections at P50. OS, outer segments; ONL, outer nuclear layer; INL, inner nuclear layer; GCL, ganglion cell layer. Scale bar, 100 µm. **B** Quantification of rod OS length in WT mice, untreated and AAV-PE treated *Pde6a* mice at P50 (n=3 eyes, three values per eye). One-way ANOVA with Tukey's multiple comparison tests. ****$p < 0.0001$. **C** Quantification of ONL thickness in WT mice, untreated and AAV-PE treated *Pde6a* mice at P50 (n=3 eyes, three values per eye). One-way ANOVA with Tukey's multiple comparison tests. ****$p < 0.0001$.

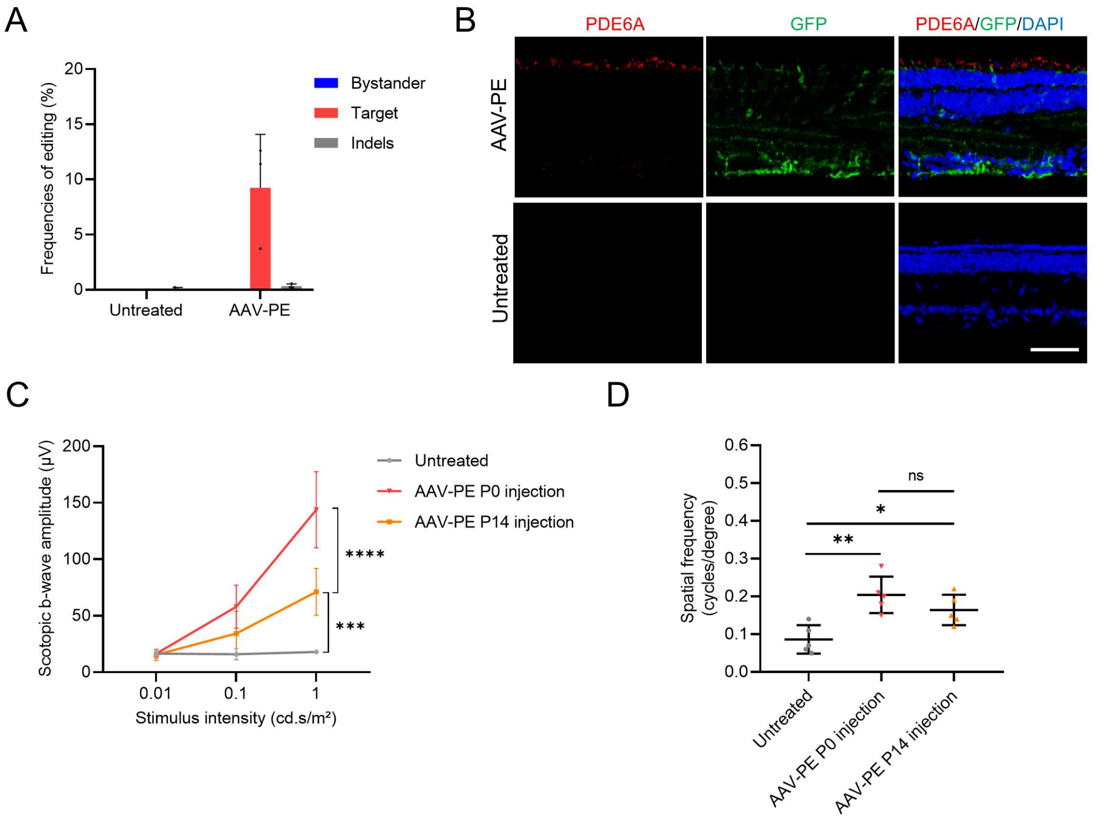

Supplementary Figure S9. AAV-PE treatment of the *Pde6a* mice by P14 injection. **A** The editing efficiency in *Pde6a* mice after AAV-PE treatment (n=3 eyes). **B** Representative immunofluorescence images of retinal sections examined with PDE6A antibody in untreated and AAV-PE treated *Pde6a* mice at P50. Nuclei were labelled with DAPI (blue). Scale bar, 50 μm. **C** Quantification of scotopic ERG b-wave amplitudes in untreated, AAV-PE P0 injection and AAV-PE P14 injection treated *Pde6a* mice at P50 (n=5 mice). Two-way ANOVA with Tukey's multiple comparison tests. ***$p < 0.001$, ****$p < 0.0001$. **D** Quantification of visual acuity in untreated, AAV-PE P0 injection and AAV-PE P14 injection treated *Pde6a* mice at P50 by OKR testing (n=5 mice). One-way ANOVA with Tukey's multiple comparison tests. *$p < 0.05$, **$p < 0.01$, ns: non-significant difference.

Supplementary Table S1. The gRNA sequences of BE and PE used in this study. Target sequence (black), PAM sequence (green).

Target site	Sequence (5’-3’)
ABE8e-nSpRY-sgRNA	ACAGACCTTGCCTTGTATTTCAA
CBE-sgRNA1	AAGGCCTGTGGCAATGATCGCGA
CBE-sgRNA2	GCAAGGCCTGTGGCAATGATCGC
PE-epegRNA	GACATCGCGATCATTGCCACAGG
PE-ngRNA1	ACACTGCTACTATTAAAGTGAGG
PE-ngRNA2	GGTTGCTGGGATTTGAACTCAGG
PE-ngRNA3	TGATCGCGATGTCCATCATGTGG
PE-ngRNA4	GGATCGCGTGCTCATGCTGCCGG
PE-ngRNA5	GCTGCCGGCGATTCAGGTTCTGG

Supplementary Table S2. Primers used for detecting target editing in this study.

Primers	Sequence (5'-3')	Product size (bp)
Pde6a-F:<br>*Pde6a*-R:	ACACTCCTGAGAGATGAGGTAG<br>CTTCAGACACACACCAGAAGAG	455
Pde6a-deepseq-F:<br>*Pde6a*-deepseq-R:	CCTTATTGCCAGCACCATTTC<br>CTTCAGACACACACCAGAAGAG	324

Supplementary Table S3. The predicted off-target sites and primers used for detecting off-target editing in this study. The mismatched bases are shown in lowercases.

Predicted off-target site		Number of mismatches	Deep sequencing primer
CBE-OT1	AAGGCCTGgGGCAATGATaG	2	F: TCTATGTCCCGCCCATAATTTC<br>R: GGTGAAGAGCTGACTCCAATATAC
CBE-OT2	AAGaaCTGTGGCAATGATgG	3	F: CAAGCCTGAGGCACCTTTAT<br>R: TACTCCCAGACTGCTTCTCTT
CBE-OT3	AAGGCCTGgGGCAATGATtt	3	F: GAGGTCGGAAGACTCTGTTATC<br>R: GGGATCCTTTGGTTTCTACCT
CBE-OT4	AAGGCCTGTGGCAATGAggt	3	F: ACCAAGGTTTGTGTCCCTATC<br>R: AACCAGAAAGTGGCAAGAAATG
CBE-OT5	AAGGtCTGTGGCtATGATgG	3	F: GGGTGAAATACCTCGCTCAA<br>R: ACACTCATACAGACATGCACATA
epeg-OT1	GACATCGCtATCcTTGCtAC	3	F: CTTGTGCTTAGCCTGCTTTC<br>R: ATCCGATGCTGTGTGTTCT
epeg-OT2	GACATCGgGATCcTTGaCAC	3	F: CCTAGCACCCAGGAAATCAA<br>R: AAACCCTAACCCTAACCCTAAC
epeg-OT3	GtCAcCGtGcTCATTGCCAC	4	F: GTTCCTAGCATCCTGCCTATG<br>R: GTGGTCCTTTCTCTGGTGATT
epeg-OT4	GcCATgGCcATCATTGaCAC	4	F: GTGCCTGACTCTCTGTTTATCTC<br>R: GGTGTCCCTGTGCTTCTTAAT
ng-OT1	ACAgTaCTACTATTcAAGTG	3	F: GCTTGCAACCTACTTGTTGTC<br>R: CCTCTGGAACCAGCCTTTAAT
ng-OT2	ACACaGaTAgTATTAAAGTG	3	F: GTGAGTTCTAAGCCAACTAGGG<br>R: AACAGTTGTCAACCACCTATCA
ng-OT3	ACACTGCTtCTAcTAAAGaG	3	F: GCAACACCTGCAGACAGTAT<br>R: ACAGTGAGTCACACAGCTTTAG
ng-OT4	tCACTaCTACTATTcAAGTG	3	F: TGGAGGAGGTAACGACTTACT<br>R: AGCCTGCTCTTGCTTTAACT

Supplementary Table S4. The ERG source data of Figure 7B and 7C.

Stimulus intensity (cd.s/m^2)	WT					Untreated					AAV-PE				
**Scotopic a-wave amplitude (µV)**															
**0.01**	**9.2**	**7.1**	**8.7**	**12.2**	**12.0**	**6.7**	**4.7**	**2.4**	**3.8**	**6.3**	**3.4**	**5.9**	**8.0**	**1.9**	**2.9**
**0.1**	**69.8**	**65.3**	**49.5**	**68.1**	**60.3**	**0.2**	**4.7**	**1.9**	**3.2**	**3.4**	**10.6**	**14.1**	**10.1**	**6.5**	**7.5**
**1**	**203.1**	**121.8**	**110.0**	**138.7**	**94.4**	**2.5**	**9.0**	**1.3**	**3.4**	**2.7**	**14.3**	**14.7**	**21.7**	**15.9**	**15.7**
**Scotopic b-wave amplitude (µV)**															
**0.01**	**254.0**	**155.5**	**132.0**	**180.3**	**194.6**	**14.7**	**21.7**	**13.3**	**19.2**	**12.8**	**12.6**	**17.9**	**14.4**	**21.9**	**15.0**
**0.1**	**367.1**	**208.0**	**190.2**	**175.4**	**253.4**	**17.6**	**11.7**	**21.7**	**9.9**	**18.6**	**73.9**	**37.7**	**68.5**	**36.6**	**73.2**
**1**	**516.0**	**290.2**	**252.3**	**330.4**	**309.9**	**20.4**	**19.2**	**16.9**	**16.0**	**17.1**	**142.7**	**94.5**	**178.9**	**170.9**	**132.0**

Supplementary references

1. R. D. Chow, J. S. Chen, J. Shen, S. Chen, A web tool for the design of prime-editing guide RNAs. Nat Biomed Eng 5, 190-194 (2021).
2. J. Y. Hsu et al., PrimeDesign software for rapid and simplified design of prime editing guide RNAs. Nat Commun 12, 1034 (2021).
3. M. V. Anderson, J. Haldrup, E. A. Thomsen, J. H. Wolff, J. G. Mikkelsen, pegIT - a web-based design tool for prime editing. Nucleic Acids Res 49, W505-w509 (2021).
4. Y. Li, J. Chen, S. Q. Tsai, Y. Cheng, Easy-Prime: a machine learning-based prime editor design tool. Genome Biol 22, 235 (2021).
5. G. Yu et al., Prediction of efficiencies for diverse prime editing systems in multiple cell types. Cell 186, 2256-2272.e2223 (2023).
6. N. Mathis et al., Predicting prime editing efficiency and product purity by deep learning. Nat Biotechnol 41, 1151-1159 (2023).

Supplementary sequence used in this study

AID:

DSLLMNRREFLYQFKNVRWAKGRRETYLCYVVKRRDSATSFSLDFGYLRNKNGCHV

ELLFLRYISDWDLDPGRCYRVTWFISWSPCYDCARHVADFLRGNPNLSLRIFTARLYF

CEAGRREPEGLRRLHRAGVQIAIMTFKDYFYCWNTFVENHGRTFKAWEGLHENSVR

LSRQLRRILL

AID-N51A: (N51A mutation, red)

DSLLMNRREFLYQFKNVRWAKGRRETYLCYVVKRRDSATSFSLDFGYLRAKNGCHV

ELLFLRYISDWDLDPGRCYRVTWFISWSPCYDCARHVADFLRGNPNLSLRIFTARLYF

CEAGRREPEGLRRLHRAGVQIAIMTFKDYFYCWNTFVENHGRTFKAWEGLHENSVR

LSRQLRRILL

Whole sequence of epegRNA4: (gRNA+scaffold+RTT+PBS+linker+evopreQ1 motif)
GACATCGCGATCATTGCCACGTTTTAGAGCTAGAAATAGCAAGTTAAAATAAGGCT
AGTCCGTTATCAACTTGAAAAAGTGGCACCGAGTCGGTGCGGCAAGGTCTGTGGC
AATGATCGCG**CCTATAAA**CGCGGTTCTATCTAGTTACGCGTTAAACCAACTAGAA

Raw Images of Figure 5F

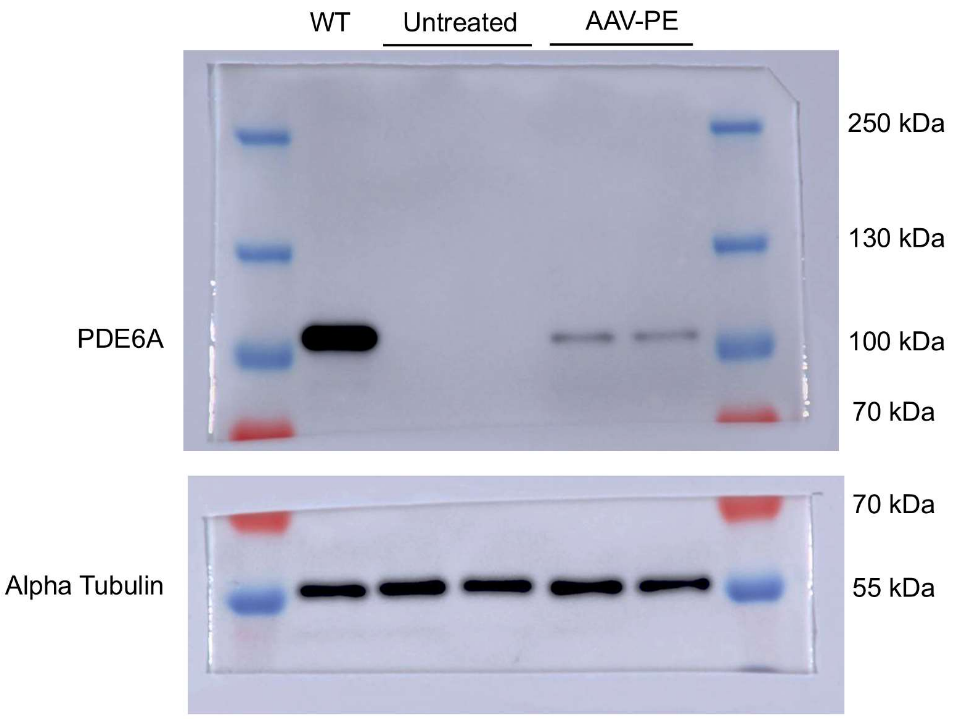

Supplement: Supplementary file 1 — Supporting Information [file ADVS-11-2405628-s001.pdf]
